# S85. THE EFFECT OF LONG-TERM SOCIAL DEPRIVATION ON EFFORT ALLOCATION PATTERN IN PATIENTS WITH SCHIZOPHRENIA

**DOI:** 10.1093/schbul/sby018.872

**Published:** 2018-04-01

**Authors:** Min-yi Chu, Hui-xin Hu, Hua Ni, Wei-hong Lu, Simon S Y Lui, Eric F C Cheung, Zhen-hui Yi, Raymond Chan

**Affiliations:** 1 Shanghai Jiao Tong University School of Medicine; 2 Shanghai Xuhui Mental Centre; 3 Shanghai Mental Health Centre, Shanghai Jiao Tong University School of Medicine; 4 Castle Peak Hospital; 6 Chinese Academy of Sciences

## Abstract

**Background:**

Motivational deficit is a common feature of negative symptoms in patients with schizophrenia. Patients with schizophrenia are impaired in goal-directed behaviour and effort allocation decision-making to pursue a potential reward. On the other hand, limited work has suggested that schizophrenia patients who experienced long-term social deprivation showed more severe negative symptoms. However, it is not yet fully clear the long-lasting impact of long-term social deprivation on motivation in these patients. The current study aimed to investigate the effect of long-term social deprivation on effort allocation pattern in patients with schizophrenia.

**Methods:**

We recruited 21 patients with schizophrenia institutionalized for more than 15 years and 20 patients with schizophrenia dwelling in the community and 24 healthy controls for this study. We administered the Effort-Expenditure for Rewards Task (EEfRT) to capture reward-based motivational salience, which requires participants make decision to choose a hard or easy task based on reward probability and magnitude. Moreover, a set of self-reported checklists including the Chapman Psychosis Proneness Scales, the Temporal Experience of Pleasure Scale, the Anticipatory and Consummatory Interpersonal Pleasure Scale and the Emotional Expressivity Scale were also administered to all the participants. For patients with schizophrenia, they also received rating score on the Positive and Negative Syndrome Scale (PANSS) and the Scale for the Assessment of Negative Symptoms (SANS).

**Results:**

Institutionalized patients had exhibited significantly more prominent negative symptoms, especially in alogia subscale, attention subscale, and a trend of statistical significance in anhedonia subscale of SANSS. The two clinical groups did not differ in positive symptoms subscale and general psychopathology symptoms subscale of PANSS. Findings from one-way ANOVA analysis showed that both institutionalized patients and community-dwelling patients with schizophrenia did not differ from healthy controls in experiential pleasure and emotion expression. For performance in the EEFRT, amotivation was only observed in institutionalized patients with schizophrenia, they were significantly less likely to expend effort to pursue a potential reward than healthy controls in both medium (50%) probability and high (80%) probability level. Hence, as the reward probability increased, unlike healthy controls, institutionalized patients could not increase their hard task choices.

**Discussion:**

Institutionalized patients with schizophrenia exhibited significantly more motivational deficits than healthy controls, and such impairment was not observed in community-dwelling patients. However, both institutionalized patients and community-dwelling patients with schizophrenia showed no deficits in self-reported scales measuring pleasure experience and expression. These findings further revealed that long-term social deprivation may be a vital contributor to severe motivation deficits of patients with schizophrenia.

